# High-grade fetal adenocarcinoma of the lung misdiagnosed as male breast carcinoma: a case report and literature review

**DOI:** 10.3389/fonc.2023.1293534

**Published:** 2023-12-06

**Authors:** Yuejian Zhuo, Yanran Xu, Rong Qin, Min Guo, Dongdong Zhang

**Affiliations:** ^1^ Department of Oncology, Xiangyang No. 1 People’s Hospital, Hubei University of Medicine, Xiangyang, China; ^2^ Department of Pathology, Xiangyang No.1 People’s Hospital, Hubei University of Medicine, Xiangyang, China

**Keywords:** fetal adenocarcinoma, male breast carcinoma, metastasis, lung cancer, poor prognosis

## Abstract

**Background and aim:**

High-grade fetal adenocarcinoma of the lung (HG-FLAC) is a specific subtype of lung adenocarcinoma with a poor prognosis. A lack of understanding exists because of the rarity of this disease. This study aimed to present a case of HG-FLAC with multiple metastases misdiagnosed as male breast carcinoma at the initial diagnosis.

**Case presentation:**

The patient visited our hospital due to a month-long cough. The chest computed tomography (CT) scan revealed a mass in the left lung and chest wall, accompanied by enlargement of mediastinal lymph nodes. The magnetic resonance imaging indicated potential metastatic lesions in the brain and adrenal glands. The patient underwent a biopsy of the lesion in the right chest wall. The pathological and immunohistochemical findings indicated a high possibility of male breast cancer. However, the clinical features did not support this diagnosis. Therefore, a CT-guided percutaneous lung biopsy was performed, and the pathological examination finally indicated HG-FLAC.

**Conclusions:**

We presented a complex yet interesting case in which HG-FLAC was misdiagnosed as male breast cancer. Our interesting case may stimulate discussions about the methods to manage patients with HG-FLAC.

## Introduction

Fetal adenocarcinoma of the lung (FLAC) is a rare subtype of lung adenocarcinoma with an invasive nature, accounting for less than 1% of primary lung adenocarcinoma (1, 2). FLAC was first described in 1982 as a “pulmonary endodermal tumor resembling fetal lung” and then named well-differentiated fetal adenocarcinoma due to its resemblance to the airway epithelium during the pseudo-glandular phase of fetal lung development. This phase is characterized by a complex glandular structure with tubules composed of glycogen-rich, nonciliated columnar or cuboidal cells (3, 4). FLAC has been categorized into low-grade fetal adenocarcinoma of the lung (LG-FLAC) and high-grade fetal adenocarcinoma of the lung (HG-FLAC) based on its clinicopathological features. The LG-FLAC usually occurs in nonsmoking female patients, whereas the HG-FLAC commonly occurs in elderly male patients with a history of heavy smoking. Histologically, LG-FLAC usually displays a pure pattern with low nuclear atypia and characteristic morule formation. In contrast, HG-FLAC often exhibits characteristic nuclear atypia and rare morule formation. About 80%–100% of HG-FLAC cases are mixed with conventional lung adenocarcinoma (CLA), large cell neuroendocrine carcinoma (LCNEC), or enteric adenocarcinoma (5–7). In this study, we presented a case of HG-FLAC with a chest wall mass that was misdiagnosed as male breast carcinoma upon the initial puncture pathological examination. The definitive diagnosis was only confirmed after a subsequent lung puncture biopsy.

## Case presentation

A 53-year-old man with a 35-year smoking history was admitted to our hospital due to a cough and dysphagia lasting about 1 month. The patient had no relevant clinical history. The physical examination revealed a subcutaneous nodule of the right chest wall approximately 1.5 cm in diameter, with a hard texture, poor mobility, and associated tenderness. Laboratory tests revealed elevated levels of the tumor markers CA199 (65.79 U/mL) and neuron-specific enolase (NSE, 18.13 ng/mL), while other laboratory parameters, such as complete blood count and hepatic and renal function tests, were within normal limits.

**Figure 1:**
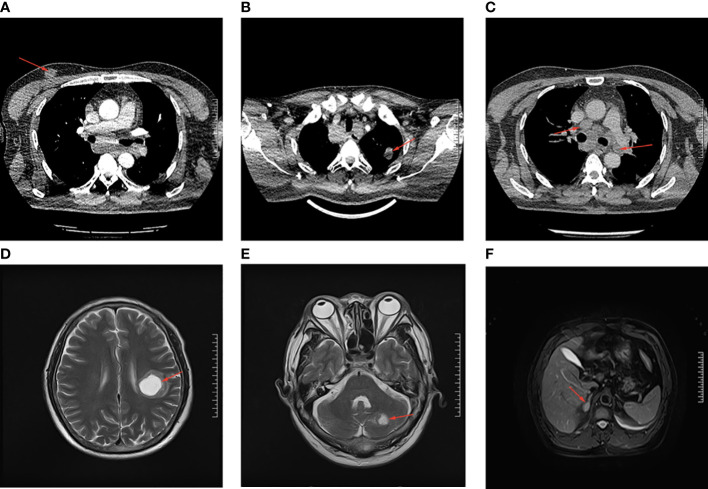
The CT and MRI imaging examination of the patient. **(A)** The subcutaneous lesion on the right chest wall. **(B)** The lobar nodule in the left upper lung. **(C)** The enlarged lymph nodes in the mediastinum. **(D, E)** The brain metastases in cerebellum and left parietal lobe. **(F)** The metastatic lesion in right adrenal. All the lesions are indicated by the red arrows.

**Figure 2:**
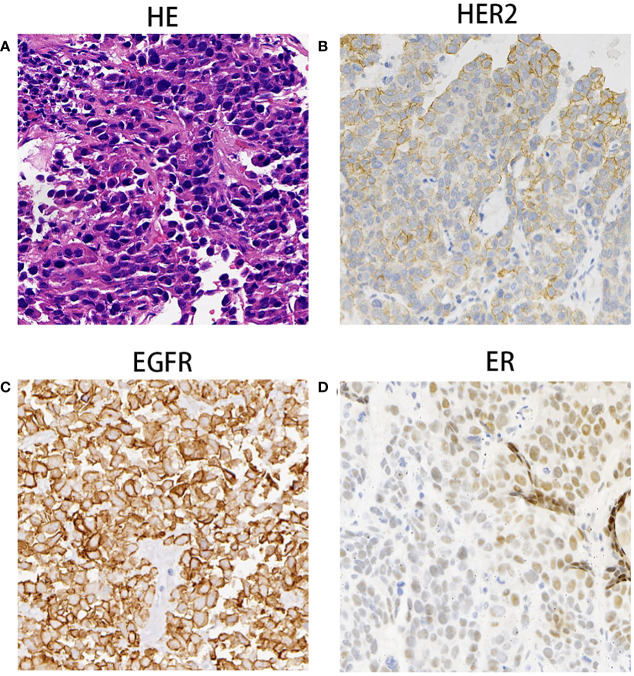
The Pathological findings of subcutaneous nodule on the right chest wall mass. **(A)** Hematoxylin and eosin staining identified complex and disorganized glandulars structures at 100× magnification. **(B–D)** Immunohistochemical analysis indicated that tumor cells were immunoreactive for HER2 (++, **B**), EGFR **(C)** and ER **(D)** at the magnification of ×100.

**Figure 3:**
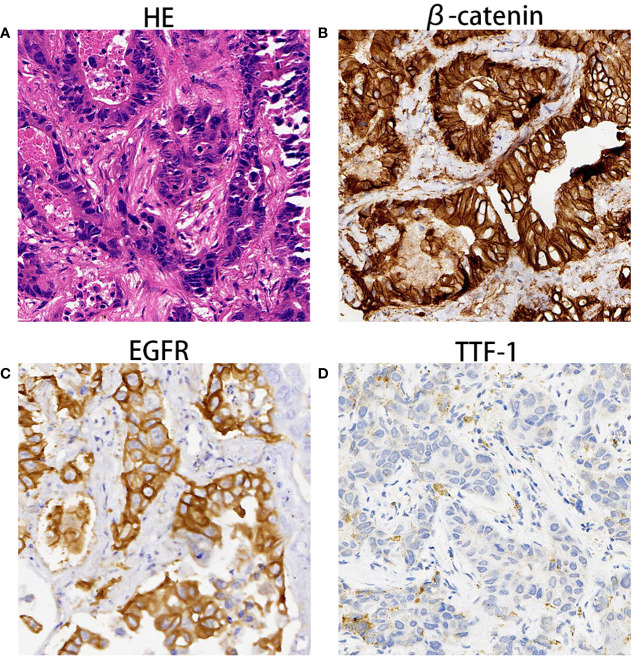
The pathological findings of lobar nodule in the left upper lung. **(A)** Hematoxylin and eosin staining showed disorganized glandulars structures resembling fetal lungs at 100× magnification. **(B–D)** Immunohistochemical analysis indicated that tumor cells were immunoreactive for β-catenin **(B)**, EGFR **(C)** and TTF1 **(D)** at the magnification of ×100.

The enhanced thoracoabdominal computed tomography (CT) scans revealed a subcutaneous mass on the right chest wall measuring 1.3 × 1.9 cm^2^ and a lobar nodule measuring 1.8 × 3.0 cm^2^ in the left upper lung. Additionally, multiple enlarged lymph nodes were observed in the bilateral clavicles, mediastinum, and left hilum. These lesions exhibited mild and inhomogeneous enhancement following the administration of iodine contrast material (**Figures 1A–C**). Further magnetic resonance imaging (MRI) examinations revealed multiple brain metastases and metastasis to the right adrenal gland (**Figures 1D–F**). Then, a needle biopsy was performed on the lesion of the right chest wall. The pathological findings suggest metastatic adenocarcinoma. Hematoxylin and eosin staining identified complex and disorganized glandular structures (**Figure 2A**). Immunohistochemical analysis revealed negative staining for thyroid transcription factor-1 (TTF-1), P63, P40, chromogranin A (CgA), and synaptophysin (Syn), but positive staining for Human epidermal growth factor receptor 2 (HER2, IHC score 2+) (**Figure 2B**), membranous epidermal growth factor receptor (EGFR) (**Figure 2C**) and estrogen receptor (ER) (more than 40%, **Figure 2D**), progesterone receptor (PR) was negative (**Supplementary Figure 1A**), suggesting the possibility of male breast cancer. However, no lesions were found in the breast area of the patient. Thus, a further CT-guided lung biopsy of the nodule at the apex of the left lung was performed. Hematoxylin and eosin staining showed complex and disorganized glandular structures resembling fetal lungs (**Figure 3A**). The immunohistochemical analysis indicated positive membranous staining for β-catenin (**Figure 3B**) and EGFR (only in the tumor cell membrane, **Figure 3C**), negative staining for TTF-1 (**Figure 3D**), alpha-fetoprotein (AFP) and P53 (**Supplementary Figures 1B, C**). Accordingly, the final diagnosis was stage IV HG-FLAC (cT1cN3M1, AJCC 8th edition staging system), accompanied by multiple distant metastases (chest wall, brain, and adrenal gland). Unfortunately, the patient refused to receive any treatment and was subsequently lost to follow-up.

## Discussion

FLAC is a histological variant of adenocarcinoma. Its classification into HG-FLAC and LG-FLAC was proposed in 1998 and formally recognized by International Association for the Study of Lung Cancer (IASLC) in 2011 (4, 8). Compared with LG-FLAC, HG-FLAC typically occurs in elderly patients with a heavy smoking history, with a more complex pathological structure and worse prognosis. In this study, we reported the case of a patient with HG-FLAC with multiple metastases initially misdiagnosed as male breast carcinoma.

HG-FLAC accounts for about 0.4%–0.5% of primary lung cancer cases. The major clinical symptoms include cough, hemoptysis, and sputum, which are nonspecific compared with conventional lung adenocarcinoma (5). HG-FLAC is more prevalent in men, particularly in elderly patients with a heavy smoking history, usually presenting in advanced stages with poor prognoses (1, 9, 10).

The definitive diagnosis of HG-FLAC mainly relies on pathological examination, although imaging modalities such as CT and MRI can provide supplementary diagnosis. Histologically, FLAC is characterized by glandular structures, with tubules composed of nonciliated, glycogen-rich cells resembling fetal lung tubules, accompanied by morule formation within the luminal space (4). Compared with LG-FLAC, HG-FLAC is characterized by a more complex and irregular glandular structure, marked nuclear atypia, and frequent mitotic activity, with morule formations rarely observed. Additionally, about 80%–100% of HG-FLAC cases exhibit mixed components of other lung cancer types, including conventional lung adenocarcinoma (CLA), large cell neuroendocrine carcinoma (LCNEC), or enteric adenocarcinoma (5). Only very few cases of pure HG-FLAC have been reported (6).

The immunohistochemical characteristics of HG-FLAC are not well established. Membranous staining for β-catenin is considered a characteristic feature. Other immunohistochemical markers, such as neuroendocrine markers (CgA, CD56, and Syn), often show positive expression in HG-FLAC. Positive staining for the embryonic marker alpha fetoprotein (AFP) was observed in 29% of cases in a previous study. However, the other two studies reported higher rates of AFP staining positivity, which were 47% and nearly 90%, respectively (4, 9, 11). Other markers, such as CDX2, were positive in 40% of cases of HG-FLAC (11). Zhang reported that all five cases of HG-FLAC showed membranous EGFR(+) (1). Further, positive staining of HER2 was reported in two cases, and the IHC score was 2 in both cases. In our case, the IHC staining score for HER2 was 2+, and the ER was strongly positive (more than 40%). Additionally, the patient in this study had chest wall metastasis, which led to misdiagnosis of male breast cancer (12, 13).

The treatment consensus for HG-FLAC has not been established yet because of the rarity of the disease. Treatment approaches should be tailored to individual patient’s conditions. Surgery remains the mainstay treatment for resectable patients, with adjuvant chemotherapy and radiation therapy as postoperative options. For unresectable or advanced-stage patients, platinum-based chemotherapy and radiation therapy can be used as optional treatment modalities. However, the effectiveness of this treatment approach is yet to be established (6, 10). Relevant studies on targeted therapy are lacking. Unlike the predominance of *EGFR* mutations in CLA, HG-FLAC exhibits distinct molecular mechanisms characterized by frequent mutations in *TP53* (44%), *KRAS* (11%–25%), and *KMT2C* (38.8%). Thus, WEE1 and KRAS G12C inhibitors can be considered as candidate therapeutic approaches (11, 14, 15). Additionally, HER2 positive was reported in our study, besides the other two reported cases, which indicated that HER2 inhibitors may offer new avenues for treatment (8, 12). Nevertheless, studies with a larger sample size are needed to confirm this hypothesis. A previous study indicated that HG-FLACs displayed low PD-L1 expression (1%–49% in 5/16 cases and <1% in 11/16 cases), suggesting a poor response to PD-L1 immunotherapy. However, Fujimoto et al. reported that a patient with PD-L1 TPS <1% received carboplatin plus nab-paclitaxel combined with atezolizumab treatment and achieved partial remission; a sustained response was observed after maintenance with atezolizumab monotherapy (12, 14). Therefore, further studies are needed to explore the potential molecular mechanism and identify effective treatment approaches. Combining multiple treatment modalities may potentially provide significant benefits to patients.

In conclusion, we reported a rare case of HG-FLAC with chest wall metastasis misdiagnosed as male breast cancer. We also synthesized the diagnosis and treatment strategy for this rare condition by reviewing the relevant studies. This study may provide some valuable insights into managing this rare disease. However, the underlying molecular mechanisms of HG-FLAC remain unclear. Hence, further exploration is needed to determine the optimal treatment strategy.

## Data availability statement

The original contributions presented in the study are included in the article/**Supplementary Material**. Further inquiries can be directed to the corresponding author.

## Ethics statement

This study was approved by the ethics and scientific committee of the Hubei University of Medicine with approval number 2022PRH002. The study was conducted in accordance with the local legislation and institutional requirements. Written informed consent for participation in this study was provided by the participants’ legal guardians/next of kin. Written informed consent was obtained for the publication of this case report.

## Author contributions

YZ: Data curation, Formal analysis, Writing – original draft. YX: Data curation, Resources, Validation, Writing – original draft. RQ: Data curation, Formal analysis, Investigation, Validation, Writing – review & editing. MG: Data curation, Formal analysis, Investigation, Resources, Software, Writing – review & editing. DZ: Conceptualization, Data curation, Funding acquisition, Project administration, Writing – review & editing.

## Supplementary material

Supplementary Figure 1 Immunohistochemical results of chest wall mass and lung nodule biopsies. **(A)** Negative immunohistochemistry staining for PR in subcutaneous nodule of chest wall. **(B, C)** Negative immunohistochemistry staining for AFP **(B)** and TP53 **(C)** in pulmonary nodule.
