# Supplementary figures and images for: High-grade fetal adenocarcinoma of the lung misdiagnosed as male breast carcinoma: a case report and literature review
Source: Front Oncol. 2023 Dec 6;13:1293534. doi: 10.3389/fonc.2023.1293534 (PMC10730678; doi:10.3389/fonc.2023.1293534)

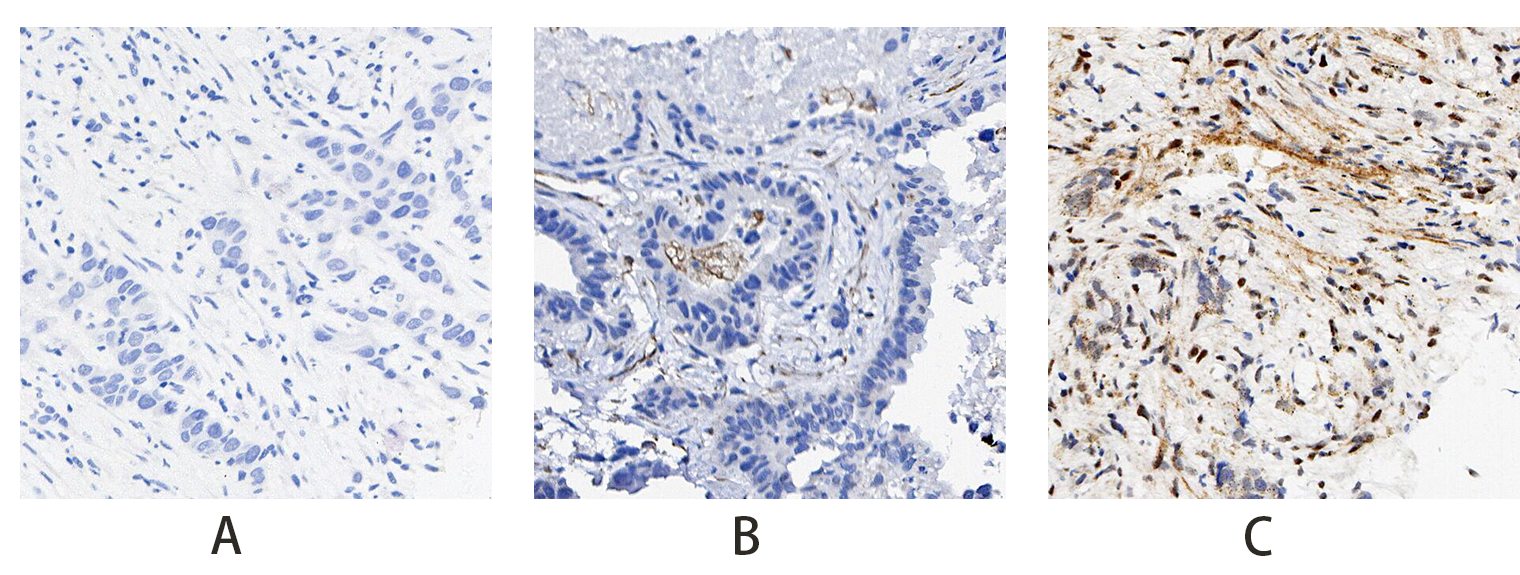

Supplement: Supplementary Figure 1 — Immunohistochemical results of chest wall mass and lung nodule biopsies. (A) Negative immunohistochemistry staining for PR in subcutaneous nodule of chest wall. (B, C) Negative immunohistochemistry staining for AFP (B) and TP53 (C) in pulmonary nodule. [file Image_1.tif]
